# Lifestyle Knowledge and Behavior Among Stroke and High-Risk Younger Adult Patients Through Sex, Age and Stroke Status Differences: A Cross-Sectional Study

**DOI:** 10.1177/15598276251343016

**Published:** 2025-05-23

**Authors:** Sarah Ibrahim, Jasper R. Senff, Troy Francis, Aleksandra Stanimirovic, Sharon Ng, Lindsey Zhang, Akshaya Ravi, Leanne K. Casaubon, Keithan Sivakumar, Joanathan Rosand, Sanjula Singh, Valeria E. Rac, Aleksandra Pikula

**Affiliations:** 1 Program for Health System and Technology Evaluation, 198139 Toronto General Hospital Research Institute, Toronto, ON, Canada (SI, TF, VER); 2 Jay and Sari Sonshine Centre for Stroke Prevention & Cerebrovascular Brain Health, 26625 Toronto Western Hospital, University Health Network, Toronto, ON, Canada (SI, VER, AP); 3 Institute of Health Policy, Management and Evaluation (IHPME), Dalla Lana School of Public Health, 7938 University of Toronto, Toronto, ON, Canada (SI, TF); 4 Centre for Advancing Collaborative Healthcare & Education, 7938 University of Toronto, Toronto, ON, Canada (SI); 5 Henry and Allison McCance Center for Brain Health, 2348 Massachusetts General Hospital, Boston, MA, USA (JRS, SN, JR, SS); 6 Department of Neurology, 2348 Massachusetts General Hospital, Boston, MA, USA (JRS, SN, JR, SS); 7 Broad Institute of MIT and Harvard, Cambridge, MA, USA (JRS, SN, JR, SS); 8 Center for Genomic Medicine, 2348 Massachusetts General Hospital, Boston, MA, USA (JRS, JR, SS); 9 Department of Neurology and Neurosurgery, Brain Center Rudolf Magnus, University Medical Center Utrecht, Utrecht, Netherlands (JRS); 10 Ted Rogers Centre for Heart Research at Peter Munk Cardiac Centre, 198139 Toronto General Hospital Research Institute (TGHRI), University Health Network (UHN) (TF, VER); 11 Harvard Chan School of Public Health, Boston, MA, USA (SN); 12 Faculty of Medicine, 6363 Ottawa University, Ottawa, ON, Canada (LZ); 13 Bloomberg School of Public Health, 1466 Johns Hopkins University, Baltimore, MD, USA (AR); 14 Department of Medicine, Division of Neurology, 7938 University of Toronto, Toronto, ON, Canada (LKC, KS, AP); 15 Department of Neurology, 26625 Toronto Western Hospital, UHN, Toronto, ON, Canada (LKC, KS, AP); 16 Krembil Brain Institute, UHN, Toronto, ON, Canada (AP)

**Keywords:** younger adults, lifestyle medicine, lifestyle behaviors, knowledge

## Abstract

**Background:** The prevalence of stroke is projected to rise over the next 30 years, particularly among younger adults (≤65 years of age). Stroke is associated with modifiable risk factors, highlighting the importance of risk factor modification. However, to modify risk factors, it is important to understand younger adult stroke and high-risk patients’ lifestyle-related knowledge, behaviors and associated facilitators and barriers, which this study aimed to address with attention to sex, age, and stroke status-related differences. **Methods:** A cross-sectional study was conducted. Data were collected through an online self-reported survey. Descriptive and inferential statistics were conducted with attention to sex, age, and stroke status differences. **Results:** A total of 104 participants comprised the sample. Variability in lifestyle-knowledge was found. Most participants ate processed food, moderately exercised, slept <7 hours per night, had a sense of social connectedness, and moderate-to-manageable stress. Emotions, social and family responsibilities influenced diet and exercise. Sex, age, and stroke status differences were observed. **Conclusions:** Findings have implications on the development of lifestyle medicine prescriptions and interventions as standard of care to support brain health and reduce the risk of stroke and/or its reoccurrence.

“Most participants were willing (98.1%) to change their lifestyle habits in the upcoming six months to improve their health.”

## Introduction

Over the past 10 years, the epidemiological shift in stroke incidence has alarmingly transformed the stroke landscape. Stroke incidences have significantly increased (∼40%) among the working population (≤65 years of age) with approximately 15% among persons ≤50 years of age^1^ and projections of prevalence rising over the next 30 years.^2^ In this population, stroke has detrimental personal (e.g., identity, family, physical, mental health), professional (e.g., careers, work), societal, health care, and economic impacts.^3,4^ Appreciating such unique impact of stroke on younger adults, compared to older adults, and the paucity of research among this patient population, the research team conducted a needs assessment study to assesses younger adult stroke patients: (1) physical, psychological, and occupational functioning and health-related quality of life; and (2) post-stroke care preferences using patient reported outcome measures.^5^ A key finding from the needs assessment study was that ≥70% of the participants (N = 85) reported the need for additional support and interventions on their physical health and preferred in-person post-stroke care led by health care providers.^5^

More importantly, the rising stroke incidences among younger adults have been strongly attributed to modifiable risk factors (MRFs).^6-9^ Further, while the literature is emerging on the recurrence of stroke among younger adults, the risk remains significant particularly for those with unmanaged risk factors such as hypertension, diabetes, and smoking.^10,11^ This highlights the importance of focusing on risk factor modification and the adoption of healthy lifestyle habits,^12,13^ which can be achieved through lifestyle medicine (LM).^14-16^ LM, the cornerstone of primary and secondary stroke prevention,^17,18^ is an evidence-based clinical discipline that applies a root-cause approach to optimize healthy lifestyle behaviors to promote health, prevent, manage and/or treat disorders and the associated MRFs.^16,19,20^ LM comprises of six pillars—nutrition, physical activity, sleep health, stress management, substance use, and social connectedness.^19^ There are countless studies supporting the positive effect of LM on reducing chronic disease (e.g., type 2 diabetes), and cerebrovascular disease (stroke) risk and reoccurrence.^16,21-23^ The strength of the LM research is underscored by numerous evidence-based clinical guidelines from the American Heart Association^24,25^ and the Heart and Stroke Foundation of Canada^26^ on risk factor modification through positive and healthy lifestyle behaviors.

According to several behavioral change theories (e.g., health belief model, knowledge attitude practice model, social cognitive theory) and empirical evidence, a precursor to behavior change is knowledge.^27-29^ While knowledge is essential to promote healthy behaviors, it is insufficient on its own^30^ and often does not translate into the adoption and sustainment of healthy behaviors.^31^ This highlights the importance of understanding the contextual facilitators and barriers to behavior change^32^ as this influences the adoption of positive and healthy behaviors. Such information is vital to apprise the design (e.g., content, mode of delivery) and successful implementation of evidence-based behavioral interventions that support, equip, as well as adequately address the contextual and influencing factors, needs and preferences of younger adults; all of which supports brain health which is vital for healthy aging, functional independence, and predicting quality of life.^33^ However, to date, lifestyle-related knowledge, behaviors and influencing factors are not well understood among younger adult stroke and high-risk patients. This study aimed to fill this gap by attaining an understanding of younger adult stroke and high-risk stroke patients’ lifestyle-related knowledge and behaviors as well as influencing factors for the adoption of healthy lifestyle behaviors with attention to also sex, age and stroke status differences.

## Methods

### Study Design and Ethical Consideration

A cross-sectional study was conducted. The study was part of a larger explanatory sequential mixed methods quality improvement (*Know Brain Embrace Care*) investigation of younger adult stroke patients’ knowledge around health risk factors, brain health and LM concepts, facilitators and barriers with the adoption of healthy lifestyle habits as well as recommendations for preferred LM-related interventions to support brain health prevention. The findings for this study are reported using guidelines of the Strengthening the Reporting of Observational Studies in Epidemiology (STROBE) for cross-sectional studies.^34^ The study was approved by the UHN Quality Improvement Committee (QIRC#23-0518). Verbal consent was attained by the research coordinator.

### Setting and Participants

The study was conducted at the Toronto Western Hospital (TWH), University Health Network (UHN) located in Toronto, Canada, which is one of the largest stroke centers in the province (Ontario), affiliated with the University of Toronto. A consecutive sampling method was employed. Specifically, patients attending the Stroke Prevention or Neurovascular Clinics at TWH were approached from June 2022-September 2023. Patients who met the inclusion criteria were approached and recruited by a research coordinator. The study population encompassed younger adult patients who at the time of participation: (1) had an ischemic or hemorrhagic stroke >90 days prior to recruitment or were at higher risk for stroke (no stroke, but a diagnosis of TIA, unruptured aneurysm or vascular malformation); (2) were of working age (≤65 years of age); (3) were receiving care at either the Stroke Prevention (stroke or TIA) or Neurovascular (aneurysm or vascular malformation) Clinic at TWH; and (4) were able to communicate in English. Patients were excluded if they had a known brain injury that resulted from trauma and had known advanced cognitive impairment (defined as a diagnosis of dementia and/or mRS >(4) that would preclude them from providing informed consent. Patients were considered for inclusion, based on assessments by members of the health care team (e.g., Neurologists, Nurse Practitioners) during clinic visits and proceeded to inform the research coordinator. Following this, potential participants were approached by the research coordinator to explain the study, provide a letter of information, and answer any study-related questions.

### Data Collection

Data were collected from the consented participants through a survey, which was either hard copy or electronic (based on the preference of the participant).

### Sociodemographic and Clinical Characteristics

Participant’s sociodemographic and clinical characteristics were collected through a REDCap survey. *Sociodemographic characteristics* were assessed with questions pertaining to sex, gender, age, level of education, ethnicity, language, marital status, employment status, family structure, and household income. C*linical characteristics* were assessed with questions about stroke status, number of strokes, time since stroke (in months) and diagnosis of any other neurological condition.

### Variables and Instrumentation

The study variables were: (1) lifestyle knowledge; (2) lifestyle status and associated facilitators and barriers; and (3) health behavior motivation.

#### Lifestyle Knowledge

Data were obtained through questions on lifestyle knowledge using an investigator-developed questionnaire that was guided by the Life’s Essential 8 recommendations^24^ and the Brain Care Score (BCS).^35,36^ All participants received multiple-choice questions with different cut-off values as options and were asked to select the recommended guidelines on blood pressure, physical activity, nutrition, substance use, sleep health, and alcohol use, with definitions and examples provided when necessary. The correct recommendations included: (i) a blood pressure of 120/80 mmHg or below; (ii) at least 150 minutes of moderate exercise per week—for example, brisk walking or activity that makes one breath harder, but can talk; (iii) consuming at least 4.5 servings of fruits and vegetables per day—for example, one serving equal to a handful or one cup and does not include fruit juice; (iv) 2 servings of lean protein per day—for example, an adult portion equivalent to about a palm size; (v) 3 servings of whole grains per day—for example, brown rice, whole wheat pasta/bread, quinoa, whole oats—unprocessed with one adult portion equivalent to one cup or one slice; (vi) 1500 mg of sodium intake per day—for example, one teaspoon of table salt has 2300 mg of sodium; (vii) a sleep duration of 7-8 hours per night; and (viii) 1-2 alcoholic drinks per day. We assessed the level of knowledge by the number of correct recommendations, ranging from 0 to 8.

#### Lifestyle Behaviors

Data were obtained through questions on participants’ lifestyle status using investigator-developed questions guided by the American Heart Association and Centres for Disease Control and Prevention recommendations and the American College of Lifestyle Medicine (ACLM) validated tools.^37^ Questions focused on all six pillars of LM and inquired about the extent to which participants engaged (e.g., frequency, duration, amount) with the various pillars. For example, (e.g., if asked about moderate physical activities, participants were given a simple definition: moderate physical activity is an activity that takes physical effort and makes you breathe somewhat harder than normal but able to talk, examples: brisk walking, carrying light load, biking at regular pace). Physical activity, specifically for walking, was converted into daily steps.^38^

#### Health Behavior Motivation

The Health Behaviour Motivation Scale (HBMS) was used. The HBMS is based on the Self-Determination Theory and measures persons’ motivation towards pro-health behaviors. The tool comprises of five dimensions: (1) intrinsic regulation; (2) identified and reintegration regulation; (3) introjected regulation; (4) external regulation; and (5) amotivation. The tool is anchored on a 5-point Likert scale ranging from 1 (statement not suiting me at all) to 5 (statement suits me very well). The HBMS is psychometrically robust, Cronbach’s α = 0.91-0.94.^39^

### Statistical Methods

Descriptive and inferential statistics were conducted using SPSS (Version 29)^40^ and R software v4.1.0.^41^ The sample was described using descriptive statistics. Lifestyle knowledge-related variables, which were categorical, were described using counts and percentages. Lifestyle status-related variables, which were continuous and categorical, were described using means and standard deviations (SDs) and counts and percentages, respectively. We employed Linear Model ANOVAs for continuous data that followed normal distribution, while Pearson chi-square tests were applied for categorical data. Descriptive subgroup analysis was also performed for sex, age (≤45 year and ≥45 years) and stroke history. All analyses used a significance level of *P* < 0.05.

#### Regression Models

We performed univariable and multivariable logistic regression models with the primary outcome being the level of knowledge based on the correct number of lifestyle knowledge answers. We dichotomized the level of knowledge at the median, resulting in two groups: (i) lower level of knowledge (≤3) and (ii) higher level of knowledge (≥4).

Variables for univariable logistic regression were selected a priori, driven by past literature shown to associate with knowledge of risk factors for brain disease.^8,42-44^ These variables included age, sex, marital status, education, employment, income level, ethnicity, history of stroke, and Canadian or foreign-born status. For the multivariable analyses, we based our choice of confounders on variables which were significantly associated with lifestyle knowledge in the univariable analysis and previously published epidemiological data.^8,42,45^ We presented our logistic regression results as odds ratios (OR) with corresponding 95% confidence intervals (CI) and *P*-values. A two-tailed *P*-value of <0.05 was considered statistically significant. We evaluated multicollinearity in each model through the variance inflation factor, while model fit was assessed using McFadden’s Pseudo-R squared. A McFadden’s R2 value between 0.2 and 0.4 indicates excellent model fits.^46^

## Results

### Participants

**Figure 1.**
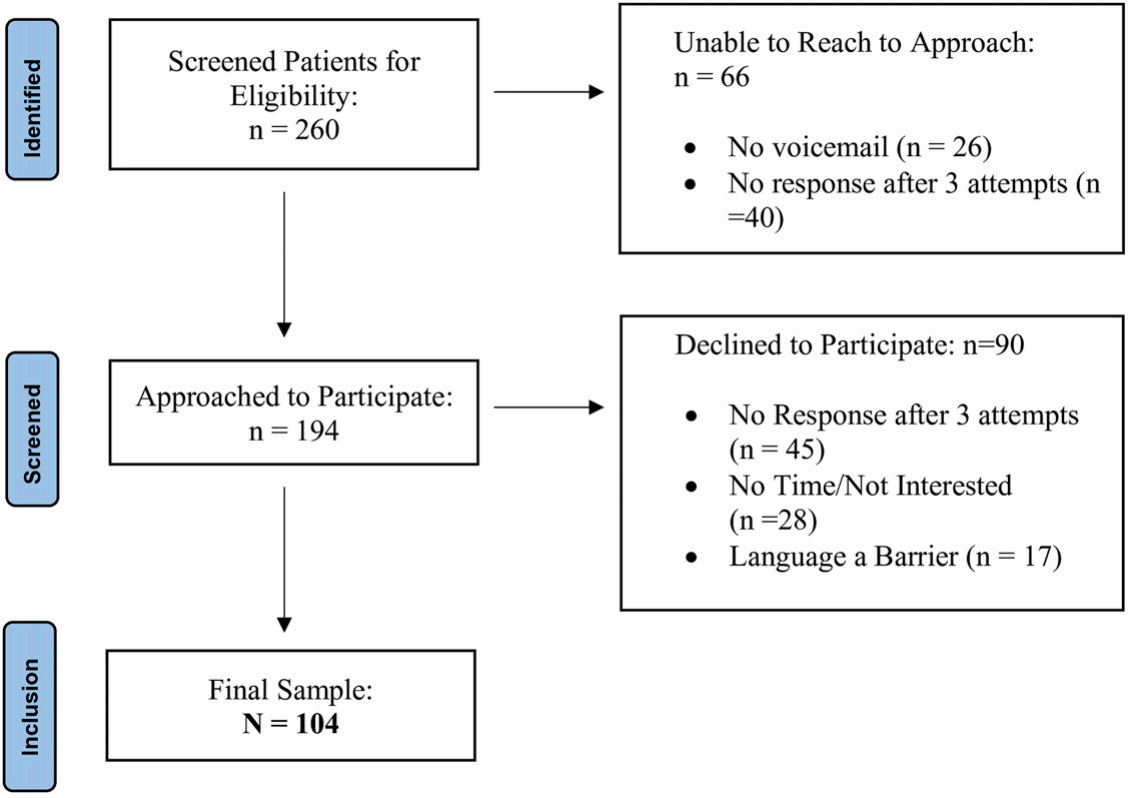
STROBE flow chart.

A total of 260 patients were screened for eligibility by a member of the health care team and of those, the research coordinator could not reach 66 to inquire about their interest to take part in the study. Of the 194 participants approached, 90 declined to take part for various reasons (e.g., no response after three attempts, lack of time and/or interest, language barrier). A total of 104 participants comprised the study sample (Figure 1).

#### Sociodemographic Characteristics

All participants except for one were cis gender. Half of the participants (52%) were female with a mean age of 47.51(SD: 14.26, range: 18-66). Most participants (59.3%) reported being white, Canadian-born (63.7%) with English as their first language (71.4%). Most participants were married/cohabitating (63.7%) and had college or university-level education (47.8%) (Table 1). Notably, there was a statistically significant difference (*P* = 0.003) with marital status for persons ≤45 year of age; with more men (75.9%) being married compared to women (45.9%). Some participants also reported working in manual (23.3%) and non-manual paid work (27.8%), and the average income varied among participants with 30.8% being $100,000-$200,000 CAD per year. There was a statistically significant difference with average income (*P* = 0.001), with persons ≤45 years having a higher income ($200,000 CAD) compared to those who were ≥45 years (supplemental Table 1).

**Table 1.** Sociodemographic Characteristics.

	Female (N = 55)	Male (N = 49)	Total (N = 104)	*P*-value
Age				0.130^b^
Mean (SD)	45.5 (15.0)	49.8 (13.2)	47.5 (14.3)	
Range	18.0 - 66.0	20.0 - 65.0	18.0 - 66.0	
Age categorized (n, %)				0.194^a^
Below 45 years	26 (47.3%)	17 (34.7%)	43 (41.3%)	
Above 45 years	29 (52.7%)	32 (65.3%)	61 (58.7%)	
Ethnicity (n, %)				0.482^a^
Missing	6	7	13	
White	26 (53.1%)	28 (66.7%)	54 (59.3%)	
Black	3 (6.1%)	1 (2.4%)	4 (4.4%)	
South Asian	2 (4.1%)	2 (4.8%)	4 (4.4%)	
East Asian	8 (16.3%)	4 (9.5%)	12 (13.2%)	
Mainland Southeast Asian	0 (0.0%)	1 (2.4%)	1 (1.1%)	
West Asian	1 (2.0%)	0 (0.0%)	1 (1.1%)	
Latin American	5 (10.2%)	2 (4.8%)	7 (7.7%)	
Arab	0 (0.0%)	1 (2.4%)	1 (1.1%)	
African	0 (0.0%)	1 (2.4%)	1 (1.1%)	
West Indian	2 (4.1%)	0 (0.0%)	2 (2.2%)	
European	2 (4.1%)	1 (2.4%)	3 (3.3%)	
Prefer not to answer	0 (0.0%)	1 (2.4%)	1 (1.1%)	
Canadian or foreign born (n, %)				0.159^a^
Missing	6	7	13	
Canadian-born	28 (57.1%)	30 (71.4%)	58 (63.7%)	
Foreign-born	21 (42.9%)	11 (26.2%)	32 (35.2%)	
Do not wish to answer	0 (0.0%)	1 (2.4%)	1 (1.1%)	
Language (n, %)				0.059^a^
Missing	6	7	13	
English second language	17 (34.7%)	7 (16.7%)	24 (26.4%)	
English first language	32 (65.3%)	33 (78.6%)	65 (71.4%)	
Do not wish to answer	0 (0.0%)	2 (4.8%)	2 (2.2%)	
Marital status (n, %)				0.110^a^
Missing	6	7	13	
Single	13 (26.5%)	16 (38.1%)	29 (31.9%)	
Married/cohabitating	32 (65.3%)	26 (61.9%)	58 (63.7%)	
Other	4 (8.2%)	0 (0.0%)	4 (4.4%)	
Level of education (n, %)				0.983^a^
Missing	6	8	14	
No degree, certificate, or diploma	2 (4.1%)	1 (2.4%)	3 (3.3%)	
High school	7 (14.3%)	5 (12.2%)	12 (13.3%)	
Some college/university	3 (6.1%)	4 (9.8%)	7 (7.8%)	
College/university	23 (46.9%)	20 (48.8%)	43 (47.8%)	
Registered apprenticeship/trade certificate	1 (2.0%)	1 (2.4%)	2 (2.2%)	
Post-graduate degree	13 (26.5%)	10 (24.4%)	23 (25.6%)	
Employment type (n, %)				0.244^a^
Missing	6	8	14	
Manual paid work	9 (18.4%)	12 (29.3%)	21 (23.3%)	
Non-manual paid work	14 (28.6%)	11 (26.8%)	25 (27.8%)	
Student	6 (12.2%)	1 (2.4%)	7 (7.8%)	
Homemaker	2 (4.1%)	0 (0.0%)	2 (2.2%)	
Unpaid volunteer	1 (2.0%)	0 (0.0%)	1 (1.1%)	
Unemployed, looking for work	3 (6.1%)	5 (12.2%)	8 (8.9%)	
On leave of absence	10 (20.4%)	4 (9.8%)	14 (15.6%)	
Retired	2 (4.1%)	4 (9.8%)	6 (6.7%)	
Disability	1 (2.0%)	3 (7.3%)	4 (4.4%)	
Not working due to illness/injury	1 (2.0%)	1 (2.4%)	2 (2.2%)	
Average income (n, %)				0.223^a^
Missing	6	7	13	
Less than $15,000	1 (2.0%)	4 (9.5%)	5 (5.5%)	
$15,000-$49,999	4 (8.2%)	6 (14.3%)	10 (11.0%)	
$50,000-$99,999	18 (36.7%)	8 (19.0%)	26 (28.6%)	
$100,000-$200,000	17 (34.7%)	11 (26.2%)	28 (30.8%)	
More than $200,000	6 (12.2%)	7 (16.7%)	13 (14.3%)	
Do not know	1 (2.0%)	1 (2.4%)	2 (2.2%)	
Do not wish to answer	2 (4.1%)	5 (11.9%)	7 (7.7%)	

^a^Pearson’s Chi-squared test.

^b^Linear Model ANOVA.

#### Clinical Characteristics

Half of the participants (53.8%) had a stroke. More males had a stroke compared to females (*P* = 0.005). The time since stroke varied among participants with a mean of 11.6 months (SD: 8.87, range: 1-24 months). Furthermore, most participants (85.1%) reported taking their prescribed medication, indicating a high medication compliance (Table 2).

**Table 2.** Clinical Characteristics.

	Female (N = 55)	Male (N = 49)	Total (N = 104)	*P*-value
Stroke occurrence (n, %)				**0.005** ^a^
No	33 (60.0%)	15 (30.6%)	48 (46.2%)	
Yes—more than one stroke	3 (5.5%)	10 (20.4%)	13 (12.5%)	
Yes—one stroke	19 (34.5%)	24 (49.0%)	43 (41.3%)	
Time since stroke diagnosis (n, %)				0.554^a^
Missing	45	33	78	
1 month ago	1 (10.0%)	0 (0.0%)	1 (3.8%)	
2-4 months ago	1 (10.0%)	5 (31.2%)	6 (23.1%)	
5-8 months ago	3 (30.0%)	4 (25.0%)	7 (26.9%)	
9-12 months ago	0 (0.0%)	1 (6.2%)	1 (3.8%)	
Greater than 1 year	1 (10.0%)	2 (12.5%)	3 (11.5%)	
Greater than 2 years	4 (40.0%)	4 (25.0%)	8 (30.8%)	
Diagnosed with other neurological disorders (n, %)				**0.011** ^a^
Missing	0	2	2	
Never	33 (60.0%)	39 (83.0%)	72 (70.6%)	
Yes	22 (40.0%)	8 (17.0%)	30 (29.4%)	
Taking medication(s) (n, %)				0.146^a^
Missing	1	2	3	
Not prescribed	6 (11.1%)	2 (4.3%)	8 (7.9%)	
No	4 (7.4%)	1 (2.1%)	5 (5.0%)	
Yes	44 (81.5%)	42 (89.4%)	86 (85.1%)	
Prefer not to answer	0 (0.0%)	2 (4.3%)	2 (2.0%)	

^a^Pearson’s Chi-squared test.

### Lifestyle Knowledge

Overall, most participants understood current lifestyle recommendations pertaining to blood pressure (79.8%), sleep (85.6%), and alcohol consumption (62.5%). However, only some were aware of the current recommendations for exercise (25%) and nutrition, specifically fruits and vegetable servings/day (40.4%), lean protein servings/day (42.3%), whole grain servings/day (14.4%), and sodium intake/day (15.4%) (Table 3).

**Table 3.** Healthy Lifestyle Knowledge.

	Female (n = 55)	Male (n = 49)	Total (N = 104)	*P*-value
Blood pressure reading (n, %)				0.541^a^
110/60 or below	4 (7.3%)	1 (2.0%)	5 (4.8%)	
**120/80 or below**	44 (80.0%)	39 (79.6%)	83 (79.8%)	
130/80 or below	6 (10.9%)	6 (12.2%)	12 (11.5%)	
130/90 or below	0 (0.0%)	1 (2.0%)	1 (1.0%)	
140/90 or below	1 (1.8%)	2 (4.1%)	3 (2.9%)	
Recommendations for moderate exercise/week (n’, %)				0.553^a^
60 minutes/week	6 (10.9%)	2 (4.1%)	8 (7.7%)	
90-120 minutes/week	9 (16.4%)	11 (22.4%)	20 (19.2%)	
120-150 minutes/week	26 (47.3%)	24 (49.0%)	50 (48.1%)	
150 or more minutes/week	14 (25.5%)	12 (24.5%)	26 (25.0%)	
Recommended fruit & vegetable serving/day (n, %)				0.001^a^
1 serving of each	3 (5.5%)	4 (8.2%)	7 (6.7%)	
2 servings of each	11 (20.0%)	19 (38.8%)	30 (28.8%)	
3 servings of each	9 (16.4%)	16 (32.7%)	25 (24.0%)	
4-5 servings of each	32 (58.2%)	10 (20.4%)	42 (40.4%)	
Recommended lean protein serving/day (n,%)				0.120^a^
1 serving of each	14 (25.5%)	22 (44.9%)	36 (34.6%)	
2 servings of each	25 (45.5%)	19 (38.8%)	44 (42.3%)	
3 servings of each	13 (23.6%)	5 (10.2%)	18 (17.3%)	
4-5 servings of each	3 (5.5%)	3 (6.1%)	6 (5.8%)	
Recommended whole grain serving/day (n, %)				0.645^a^
1 serving of each	20 (36.4%)	22 (44.9%)	42 (40.4%)	
2 servings of each	21 (38.2%)	19 (38.8%)	40 (38.5%)	
3 servings of each	10 (18.2%)	5 (10.2%)	15 (14.4%)	
4-5 servings of each	4 (7.3%)	3 (6.1%)	7 (6.7%)	
Recommended sodium intake/day (n,%)				0.209^a^
1000 mg	20 (36.4%)	26 (53.1%)	46 (44.2%)	
1200 mg	14 (25.5%)	9 (18.4%)	23 (22.1%)	
1500 mg*	12 (21.8%)	4 (8.2%)	16 (15.4%)	
2000 mg	8 (14.5%)	8 (16.3%)	16 (15.4%)	
2500 mg	1 (1.8%)	2 (4.1%)	3 (2.9%)	
Recommended sleep/night (n, %)				0.207^a^
5-6 hours	1 (1.8%)	0 (0.0%)	1 (1.0%)	
6-7 hours	2 (3.6%)	7 (14.3%)	9 (8.7%)	
7-8 hours*	49 (89.1%)	40 (81.6%)	89 (85.6%)	
Greater than 8 hours	3 (5.5%)	2 (4.1%)	5 (4.8%)	
Recommended alcoholic drinks/day (n, %)				0.679^a^
1-2 per day	35 (63.6%)	30 (61.2%)	65 (62.5%)	
3-4 per day	2 (3.6%)	3 (6.1%)	5 (4.8%)	
4-5 per day	0 (0.0%)	1 (2.0%)	1 (1.0%)	
None	18 (32.7%)	15 (30.6%)	33 (31.7%)	

^a^Linear Model ANOVA.

#### Level of Knowledge

The distribution of knowledge levels was as follows: 0 participants had only 1 correctly answered recommended guideline questions, 9 (9.7%) participants had 2 correct, 26 (25.0%) had 3, 32 (30.8%) had 4, 25 (24.0%) had 5, 10 (9.6%) had 6, 1 (1.0%) had 7, and 1 (1.0%) had 8. The median level of total knowledge was 4 (IQR 3-5).

### Lifestyle Behaviors

#### Nutrition

Overall, 45.6% participants reported eating processed food (e.g., fast food, sugary drinks) in the past two weeks, and only 18.4% consumed the recommended servings of fruits and vegetables on an average day. Feelings of sadness (34.6%), anxiety (38.5%), fatigue (34.6%), social or family situations (38.5%), boredom (31.7%), and stress (51.9%) influenced participants eating habits (Table 4).

**Table 4.** Eating Lifestyle Status.

	Female (n = 55)	Male (n = 49)	Total (N = 104)	*P*-value
Eating fast food, sugary drinks or packaged food in last 2 weeks (n, %)				0.139^a^
Missing	0	1	1	
Not at all	14 (25.5%)	20 (41.7%)	34 (33.0%)	
Several days	25 (45.5%)	22 (45.8%)	47 (45.6%)	
More than half the days	7 (12.7%)	2 (4.2%)	9 (8.7%)	
Nearly every day	9 (16.4%)	4 (8.3%)	13 (12.6%)	
Consumption of fruits and vegetable servings on average day (n, %)				0.149^a^
Missing	0	1	1	
Less than 2 servings	13 (23.6%)	17 (35.4%)	30 (29.1%)	
2-3 servings	24 (43.6%)	24 (50.0%)	48 (46.6%)	
4-5 servings	13 (23.6%)	6 (12.5%)	19 (18.4%)	
More than 5 servings	5 (9.1%)	1 (2.1%)	6 (5.8%)	
Factors impacting eating habits (n, %)				
Don’t like healthy food	5 (9.1%)	3 (6.1%)	8 (7.7%)	0.571^a^
Nutrition labels	24 (43.6%)	18 (36.7%)	42 (40.4%)	0.474^a^
Rely on packaged or fast food	14 (25.5%)	5 (10.2%)	19 (18.3%)	**0.045** ^a^
Prepare meals at home	47 (85.5%)	42 (85.7%)	89 (85.6%)	0.970^a^
Negative relationship to food	10 (18.2%)	3 (6.1%)	13 (12.5%)	0.063^a^
No time	7 (12.7%)	6 (12.2%)	13 (12.5%)	0.941^a^
Live alone or eat alone	11 (20.0%)	9 (18.4%)	20 (19.2%)	0.833^a^
Following situations or emotions impacting eating habits (n, %)				
Sadness	24 (43.6%)	12 (24.5%)	36 (34.6%)	**0.040** ^a^
Pain	8 (14.5%)	6 (12.2%)	14 (13.5%)	0.732^a^
Insomnia	10 (18.2%)	5 (10.2%)	15 (14.4%)	0.248^a^
Anxiety	29 (52.7%)	11 (22.4%)	40 (38.5%)	**0.002** ^a^
Fatigue	24 (43.6%)	12 (24.5%)	36 (34.6%)	**0.040** ^a^
Social or family situations	26 (47.3%)	14 (28.6%)	40 (38.5%)	**0.050** ^a^
Boredom	23 (41.8%)	10 (20.4%)	33 (31.7%)	**0.019** ^a^
Stress	33 (60.0%)	21 (42.9%)	54 (51.9%)	0.081^a^
None of the above	7 (12.7%)	16 (32.7%)	23 (22.1%)	**0.015** ^a^

^a^Pearson’s Chi-squared test.

Linear Model ANOVA.

#### Physical Activity

Participants reported engaging in varying levels of PA. Specific to *moderate levels*^47^ *of PA*, referred intense exercise with a heart rate between 50%–70% of maximum heart rate,^47^ participants’ mean days of engagement was 3.46 (SD: 2.40) with more than half (55.9%) reporting >30 minutes on those days. Specific to *vigorous levels* of PA, referred to 70%–85% of maximum heart rate,^47^ the mean days of engagement was 2.55 (SD: 2.10) with some (45.7%) reporting >30 minutes of PA on those days. Participants reported walking for at least 10 minutes/week for a mean of 6 days (SD 2.26) with most (69.1%) walking for >30 minutes/day and some walking 5490 and 7320 steps/day (36.2% and 33%, respectively). Barriers that limited participant’s ability to exercise centered around work responsibility (24.0%), family responsibility (21.2%), time (28.8%), energy (36.5%), and safety concerns (29.8%) (Table 5).

**Table 5.** Physical Activity Lifestyle Status.

	No Stroke (n = 48)	Had a Stroke (n = 56)	*P*-value
Days engaging in vigorous physical activity/week			0.886^a^
Missing	1	0	
Mean (SD)	2.40 (2.31)	2.46 (1.92)	
Days engaging in moderate physical activity/week			**0.043** ^a^
Missing	1	0	
Mean (SD)	2.94 (2.27)	3.89 (2.43)	
Days walking for at least 10 min. /week			**0.022** ^a^
Missing	2	0	
Mean (SD)	5.48 (2.40)	6.50 (2.04)	
Walking steps (n, %)			0.279^b^
Missing	8	2	
1830 steps	13 (32.5%)	16 (29.6%)	
5490 steps	11 (27.5%)	23 (42.6%)	
7320 steps	16 (40.0%)	15 (27.8%)	
Barriers or challenges that limit exercise (n, %)			
No barriers	11 (22.9%)	15 (26.8%)	0.821^b^
Depression	8 (16.7%)	4 (7.1%)	0.217^b^
Work responsibility	14 (29.2%)	11 (19.6%)	0.357^b^
Family responsibility	13 (27.1%)	9 (16.1%)	0.229^b^
Cost	5 (10.4%)	1 (1.8%)	0.093^b^
Time	14 (29.2%)	16 (28.6%)	1.000^b^
Fear	6 (12.5%)	3 (5.4%)	0.296^b^
Energy	18 (37.5%)	20 (35.7%)	1.000^b^
Safety concerns	18 (37.5%)	13 (23.2%)	0.135^b^
Other (e.g., undergoing chemo, recent surgery, mood, pain)	5 (10.4%)	7 (12.5%)	0.770^b^

^a^Linear-Model ANOVA.

^b^Pearson’s Chi-squared test.

#### Sleep Health

Overall, half of the participants (51.9%) reported having <7 hours of sleep/night. Few participants (39.8%) reported always being on their screen two hours before sleeping time, and most participants (75.7%) reported never using a sleeping aid to fall asleep.

#### Social Connectedness

Most participants (80.6%) described their social relationships as having at least two persons, other than their family members, that they were close with and most (63.1%) indicated always having persons who care about them. Most participants (84.5%) reported feeling that their life has a meaning and/or purpose.

#### Stress Management

Participants reported having varying levels of stress with most being manageable (48.5%) and moderate (36.9%). Sometimes (39.8%) participants felt unable to control important things in their life yet, felt fairly confident (31.1%) and very confident (31.1%) in handling personal challenges. Sometimes (38.8%) participants felt overwhelmed, most (56.3%) had little interest doing things, and feeling down, depressed or hopeless (56.3%). Few (44.7%) felt nervous, anxious or on edge on several days as well as bothered by worrying too much (46.1%) in the past two weeks. Participants employed various coping strategies: meditation (23.1%), PA (58.7%), counseling (25%), socializing with friends and family (62.5%), eating too much or too little (41.3%), and TV and/or video games (59.6%).

#### Substance Use

Most participants drank zero to one drink per week (79.6%) and never smoked (73.8%) or used recreational drugs (97.1%) and marijuana (79.6%). Few participants (14.4%) reported receiving counseling on nutrition.

### Lifestyle Adoption Around Brain Health and Motivations

Overall, most participants were willing (98.1%) to change their lifestyle habits in the upcoming six months to improve their health. The priority areas identified by participants to improve their health centered around exercise, nutrition, mental health, and sleep (Supplemental Table 2). Participants rated various reasons for engaging in a healthy lifestyle such as feeling of happiness, overall importance, guilt or remorse if health is neglected, and to make others happy (Supplemental Table 3).

### Sex, Age and Stroke-Related Differences

#### Lifestyle Knowledge

There was a statistically significant sex difference in nutrition-related knowledge. Specifically, *females* had more knowledge of recommended fruit and vegetable servings (*P* = 0.001) compared to males. There was also a statistically significant age difference in knowledge of recommended lean protein servings/day (*P* = 0.048): ≤45 years had more knowledge compared to persons ≥45 years. Likewise, a statistically significant difference was found related to physical activity (PA)-related knowledge (*P* = 0.022): those who had a stroke understood the current PA recommendations more compared to those that did not have a stroke.

### Lifestyle Status

#### Nutrition

There was a statistically significant age difference with the frequency of processed food consumption (*P* = 0.045): participants ≤45 years consumed more processed food than those ≥45 years. There was also a statistically significant sex difference with factors influencing eating habits: more females relied on packaged or fast food and had a significantly negative relationship with food compared to males (*P* = 0.045) (Table 3). There were also notable sex and age-related differences with situations or emotions influencing participants’ eating habits. Of note, sadness (*P* = 0.040), anxiety (*P* = 0.002), fatigue (*P* = 040), social and family situations (*P* = 0.050) and boredom (*P* = 0.019) influenced female’s eating habits more than males (Table 3). Similarly, sadness (*P* = 0.004), anxiety (*P* = 0.040), and boredom (*P* = 0.032) influenced the eating habits of participants ≥45 years compared to ≤45 years.

#### Physical Activity and Sleep Health

Participants who had a prior stroke engaged in more moderate PA days/week (*P* = 0.043) and walked more (days/week) for at least 10 minutes/week (*P* = 0.022) compared to those without a stroke. Also, participants ≤45 years reported always having screen time two hours before sleeping compared to ≥45 years (*P* = 0.002).

#### Stress Management and Substance Use

Sex, age, and stroke status-related differences were found with coping strategies. *For sex*, more females used counseling/psychotherapy (*P* = 0.017) as a coping strategy compared to males. *For age*, those ≤45 years used counseling (*P*=< 0.001), eating too much or too little (*P* = 0.044), and substance use (*P* = 0.031) compared to ≥45 years to cope with stress. Whereas participants ≥45 years used spirituality/faith (*P* = 0.054) as a coping strategy compared to ≤45 years. *For stroke status*, those who had a stroke used more exercise (*P* = 0.005) to cope with stress compared to those who did not have a stroke. Age differences were also found with alcohol consumption per week (*P* = 0.039): those ≥45 years consumed 2-3 drinks/week compared to ≤45 years; and participants ≤45 years consumed four or more drinks/week compared to ≥45 years.

### Regression Analysis on Knowledge Level

A univariable logistic regression showed that males were less likely to have a higher level of knowledge (OR: 0.26 [95% CI: 0.11-0.58], *P* = 0.001) than females (Table 6). Those with a post-graduate education were more likely to have a higher level of knowledge (OR: 5.43 [95% CI: 1.30-26.31], *P* = 0.03) compared to those with a maximum of a high school degree, and those with non-manual paid work were more likely to have a higher level of knowledge (OR: 3.43 [95% CI: 1.03 - 12.29], *P* = 0.05) compared to those who perform manual paid work. Table 6 reports the multivariable logistic regression performed for the level of knowledge. When controlling for confounders (age, education, and employment type), males were 71% less likely to have a higher level of knowledge compared to females (OR: 0.29 [95% CI: 0.09-0.80], *P* = 0.02). Post-graduates (OR: 8.6 [95% CI: 1.58 - 58.02], *P* = 0.02) had greater odds of having higher lifestyle knowledge when accounting for age, sex, and employment type.

**Table 6.** Multivariable Logistic Regression and Level of Lifestyle-Related Knowledge.

Variable	OR (95% CI)	*P* Value
Level of knowledge		
Sex		
Female	Reference	-
Male	0.29 (0.09–0.80)	**0.02**
Age		
Continuous	1.03 (0.99 – 1.08)	0.15
Education		
Highschool	Reference	-
College/university	2.37 (0.58 – 10.76)	0.24
Post-graduate	8.6 (1.58 – 58.02)	**0.02**
Employment type		
Manual paid work	Reference	-
Non-manual paid work	3.22 (0.84–13.26)	0.09

McFadden Pseudo *R*^2^ = 0.34.

Variance inflation factor: Sex = 1.13; Age = 1.44; Education = 1.26; Employment = 1.73.

## Discussion

Understanding the health-related knowledge and behaviors of younger adult stroke patients is warranted as its adoption and adherence is a greatest challenge.^48^ The findings from this study, the first to our knowledge conducted in Canada for this unique patient population, highlight variable levels of lifestyle-related knowledge and the complexity of adopting such behaviors, with apparent and variable facilitators and barriers.

## Lifestyle Knowledge

In general, most study participants understood the current recommendations for blood pressure, sleep and alcohol consumption, while only few were aware of nutrition and exercise-related recommendations as the two most impactful lifestyle pillars relevant to stroke prevention. Importantly, the variation in lifestyle-related knowledge is consistent with the general and stroke literature.^49-53^ As illustrated in a cross-sectional study (N = 411, mean age: 52.4. SD:7.3, range 40-74 years) of Singaporean Chinese,^49^ most (88%) participants identified one correct stroke risk factor, however, 20% were unable to name ≥1 established stroke risk factor.^49^ Similarly, in a study that was part of a prospective Norwegian Stroke-Paths of Treatment cohort,^52^ participant’s (N = 282, mean age: 71.8, 57.1% men) self-reported symptom-related knowledge increased at three months (*P* < .001) and continued at 12 months post-stroke, however there were variations in participant’s stroke risk factor-related knowledge.^52^ This is in parallel with findings from a cross-sectional study of 101 participants in Saudi Arabia (50% ≤ 65 years old, 58.4% men, two thirds overweight or obese),^53^ where only 50% of participants correctly identified stroke risk factors; while 67.4% did not think it was important to maintain a healthy weight; 66.3% did not think exercise was important; 47.8% (of those who smoked) did not feel it was important to quit, and in contrary, 63.4% felt it was important to eat healthy.^53^ Variability with lifestyle knowledge may help explain the globally emerging evidence of the rising prevalence of stroke among younger adults with attributions to MRFs such as diet and physical inactivity.^54^

A potential explanation for the variability in lifestyle-related knowledge could be explained by the public health campaigns^55,56^ that may be different in various geographic regions and/or interventions with a strong focus on some risk factors (substance use, smoking and hypertension)^57-59^ and little focus on others (nutrition, sleep, stress, physical activity, and social connectedness). These campaigns, which are delivered through different platforms (e.g., websites, social media, mass media campaigns, mobile applications), are used to reach wider audience in diverse settings (community, workplace, and hospitals, clinics)^60^ to enhance knowledge about risk factors, and non-communicable diseases.^61^ However, such campaigns are not necessary sustainably geared towards a high-risk population or for patients with prior strokes, and certainly not delivered in consistent form via clinical practice or formal medical education.^16,62^ Further, as seen in this study, having the knowledge about healthy lifestyle does not necessary translate into practicing such behaviors.^30^

## Lifestyle Behavior

In this study, we also observed inconsistent levels of healthy lifestyle behaviors among the younger adult stroke participants, although most reported willingness to change their lifestyle. These findings are consistent with the general and stroke-related literature.^50,51^ For instance, in a cross-sectional study of 333 stroke patients (mean age 62.43, SD: 10.45) in Canada,^51^ only 38% (n = 128) had intention to exercise post-discharge with living situation (F = 5.416, *P* = 0.001), monthly income (F = 49.711, *P* < 0.001), subjective norm (*r* = 0.613, *P* < 0.001), attitude (r = 0.739, *P* < 0.001) and behavioral control (*r* = 0.765, *P* < 0.001) being significant predicators of such intention.^51^ Similar findings were found in the National Health and Nutrition Examination Surveys that explored trends of unhealthy lifestyle factors among persons who had a stroke in the United Stated from 1999 to 2018.^50^ There was an overall increase in unhealthy lifestyle habits among participants (N = 2017; grouped as 20-44, 45-64 and ≥65 years), with significant increase in alcohol consumption (39.3% (95% Confidence Interval (CI): 29.8, 48.7) to 57.4% (CI: 45.7, 69.0) *P* = 0.008) and obesity (39.2% (CI: 28.3, 50.2) to 49.4% (CI: 38.9, 59.8) *P* = 0.029).^50^ Of note, participants who were separated, divorced, widowed or unemployed reported overall higher risks of multiple unhealthy lifestyle behaviors.^50^ Similarly, the consumption of unhealthy eating habits (e.g., ultra-processed food) is linked with prolonged screen time (e.g., 3 hours/day)^63^ and replacing 30 minutes of socializing, streaming or gaming with 30 minutes of physical activity was associated with a lower follow-up Body Mass Index z-score.^64^

In this study, we observed some sex and stroke-status related differences, specific to adoption of healthy lifestyle behaviors. Such variances were also noted in chronic disease-related literature. *Specific to sex*, lack of time due to competing demands of family and domestic responsibilities as well as low energy were found to influence lifestyle changes in women (N = 32, 20-50 years of age) with gestational diabetes mellitus.^65^ Similar findings were found in a qualitative study that explored motivations and barriers on healthy lifestyle among middle-aged Iranian women.^66^ One of the themes pertained to women’s responsibilities in both the family and society was time for, that interfered with their ability to adopt healthy behaviors despite having the knowledge and motivation to do so.^66^ *Specific to stroke status*, compared to non-stroke patients, stroke patients are also exposed to more lifestyle-related education and interventions as part of post-stroke rehabilitation or discharge programs, which has been found to positively influence their lifestyle knowledge and behaviors (e.g., reducing MRFs such as blood pressure, weight, and cholesterol).^67,68^

## Implications

The study findings have several notable implications. First, it is imperative to integrate LM educational interventions as part of “standard of care” for all patients, and specifically post-stroke. Integrating such interventions as “standard of care” would support the much needed and overdo paradigm shift from reaction to prevention in health care systems.^48,69^ Such interventions should also account for various barriers that negatively impact healthy lifestyle behavior changes (e.g., time, fear, personal and professional responsibilities) and integrate strategies accordingly (e.g., making exercise or physical movement a family activity). Second, although most of this study population identified as White, it is essential to ensure educational and behavioral interventions are culturally tailored. This is because stroke is a condition of disparities with ethnic, racial, sex, and gender inequities in prevalence, incidence, treatment, health care access and outcomes.^70,71^ This is further coupled with a decrement in the translation of evidence-based preventive interventions to specific communities and structurally marginalized populations^72,73^ and this may negatively impact lifestyle-related knowledge and the adoption of healthy behaviors. Third, although not fully explored in this study, it is important to focus on mental-emotional health of younger adults in clinical practice, assessments, and LM interventions as it can impact lifestyle habits (e.g., eating habits and coping strategies) and in turn, the risk of chronic conditions.^74^ Finally, the importance for integrating LM prescriptions by physicians and/or nurse practitioners to support and encourage enactment of healthy lifestyle behaviors (e.g., physical activity, healthy eating habits, reducing substance use)^75^ among younger adult stroke patients. Simply, informing patients to engage in healthy lifestyle behaviors is often too vague and unhelpful^76^ for patients. As such, providing prescriptions that are customized by accounting for the patients’ preferences and circumstances as well as designed using the elements of the SMART (Specific, Measurable, Achievable, Realistic, Timebound) objectives better supports younger adult stroke patients in achieving real, practical and incremental lifestyle changes.^76^

## Limitations

The study has several limitations. First, this was also a cross-sectional study so no causal interference can be made given that exposure and outcome are measured at the same time. Second, the inclusion criteria were limited to persons who were able to communicate in English. Third, most participants identified as being White and were of higher socioeconomic status (based on their reported income). This may limit our ability to generalize the study findings to other younger adult stroke patients who are unable to communicate in English, are of diverse cultural and ethnic background and lower socioeconomic status who may have varying level of healthy knowledge and behaviors. Fourth, one participant (0.009%) identified as non-binary. As such, a sex analysis was performed to minimize the risk of Type 1 errors with conducting a gender analysis. Fourth, the study was conducted in a single academic health science centre in Toronto, Canada and this may impact the generalizability of the study findings. Finally, self-reported surveys in stroke and identified risk(s) have limitations compared to objective measurements, as they may have risk information.^77^

## Conclusion

**Figure 2.**
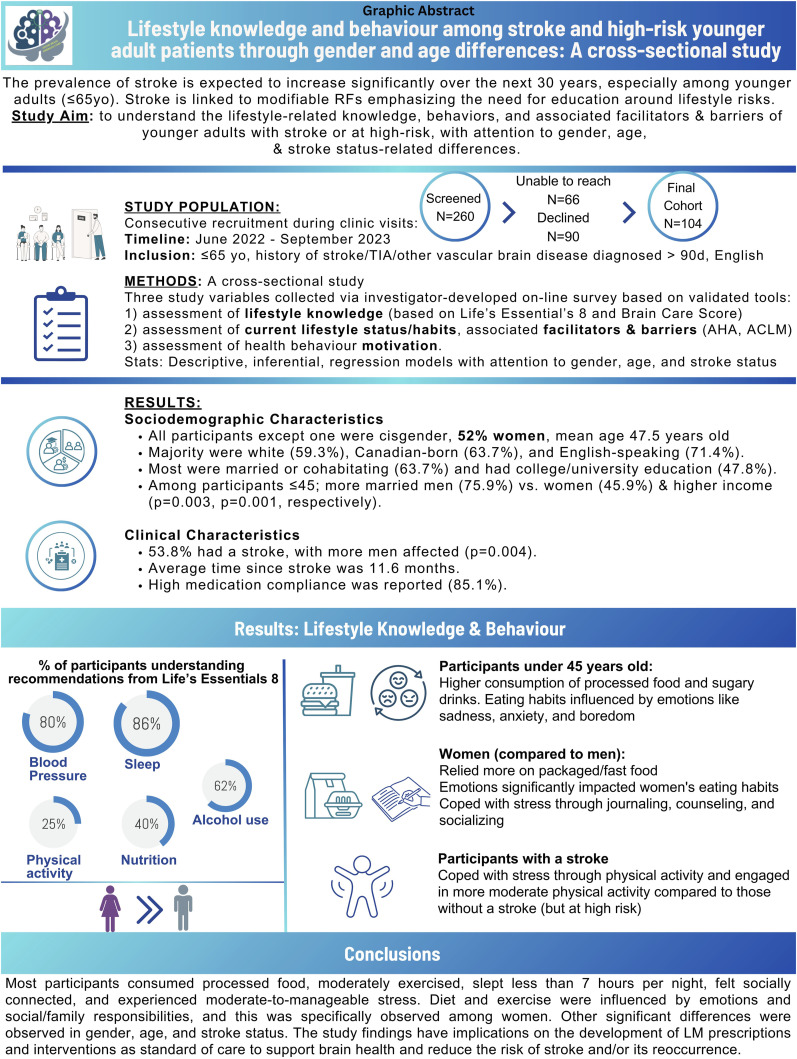
Graphic abstract of study and findings.

The global burden of stroke and its risk factors landscape has considerably changed. Increasing stroke incidences among younger adults are commonly attributed to MRFs. Abating MRFs can be achieved through, which requires lifestyle-related knowledge and the adoption of positive and healthy behaviors. However, the current lifestyle-related knowledge, behaviors and influencing factors among younger adults is not well understood. Variability in LM-related knowledge (particularly for nutrition and PA) and the adoption of healthy habits was observed in this study with sex, age, and stroke-status related differences. The study findings have implications to clinical practice and the development of fast emerging LM interventions and prescriptions to support the adoption of healthy lifestyle behaviors to support brain health and reduce the risk of stroke and/or its reoccurrence (Figure 2).

## Supplemental Material

**Supplemental Material -** Lifestyle Knowledge and Behavior Among Stroke and High-Risk Younger Adult Patients Through Sex, Age and Stroke Status Differences: A Cross-Sectional Study Supplemental Material for Lifestyle Knowledge and Behavior Among Stroke and High-Risk Younger Adult Patients Through Sex, Age and Stroke Status Differences: A Cross-Sectional Study by Sarah Ibrahim, Jasper R. Senff, Troy Francis, Aleksandra Stanimirovic, Sharon Ng, Lindsey Zhang, Akshaya Ravi, Leanne K. Casaubon, Keithan Sivakumar, Joanathan Rosand, Sanjula, Singh, Valeria E. Rac, and Aleksandra Pikula in American Journal of Lifestyle Medicine.

## Data Availability

Available upon request.*

## References

[1] Boot E Ekker MS Putaala J Kittner S De Leeuw FE Tuladhar AM . Ischaemic stroke in young adults: a global perspective. J Neurol Neurosurg Psychiatry. 2020;91(4):411-417. doi:10.1136/jnnp-2019-322424 32015089

[2] Joynt Maddox KE Elkind MSV Aparicio HJ , et al. Forecasting the burden of cardiovascular disease and stroke in the United States through 2050—prevalence of risk factors and disease: a presidential advisory from the American heart association. Circulation. 2024;150(4):e65-e88. doi:10.1161/CIR.0000000000001256 38832505

[3] Ignacio KHD Diestro JDB Medrano JMM , et al. Depression and anxiety after stroke in young adult Filipinos. J Stroke Cerebrovasc Dis. 2022;31(2):106232. doi:10.1016/j.jstrokecerebrovasdis.2021.106232 34875539

[4] Tan E Gao L Collier JM , et al. The economic and health burden of stroke among younger adults in Australia from a societal perspective. BMC Public Health. 2022;22(1):218. doi:10.1186/s12889-021-12400-5 35114974 PMC8811989

[5] Ibrahim S Francis T Sheehan KA , et al. Exploring unmet needs and preferences of young adult stroke patients for post-stroke care through PROMs and gender differences. Front Stroke. 2024;3:1386300. doi:10.3389/fstro.2024.1386300 PMC12802803 41542270

[6] Zlokovic BV Gottesman RF Bernstein KE , et al. Vascular contributions to cognitive impairment and dementia (VCID): a report from the 2018 national heart, lung, and blood institute and national institute of neurological disorders and stroke workshop. Alzheimer's Dement. 2020;16(12):1714-1733. doi:10.1002/alz.12157 33030307

[7] Livingston G Huntley J Sommerlad A , et al. Dementia prevention, intervention, and care: 2020 report of the Lancet Commission. Lancet. 2020;396(10248):413-446. doi:10.1016/S0140-6736(20)30367-6 32738937 PMC7392084

[8] Dukelow T Lawrence EG Jacobson L , et al. Modifiable risk factors for dementia, and awareness of brain health behaviors: results from the five lives brain health Ireland survey (FLBHIS). Front Psychol. 2023;13:1070259. doi:10.3389/fpsyg.2022.1070259 36710802 PMC9879702

[9] O’Donnell MJ Chin SL Rangarajan S , et al. Global and regional effects of potentially modifiable risk factors associated with acute stroke in 32 countries (INTERSTROKE): a case-control study. Lancet. 2016;388(10046):761-775. doi:10.1016/S0140-6736(16)30506-2 27431356

[10] Verburgt E Hilkens NA Ekker MS , et al. Short-term and long-term risk of recurrent vascular event by cause after ischemic stroke in young adults. JAMA Netw Open. 2024;7(2):e240054. doi:10.1001/jamanetworkopen.2024.0054 38376841 PMC10879951

[11] Malek R Alasiri S Wolfe CDA Douiri A . Major vascular events after first incident stroke: a population-based study. BMJ Neurol Open. 2024;6(2):e000723. doi:10.1136/bmjno-2024-000723 PMC11529573 39493674

[12] Boehme AK Esenwa C Elkind MSV . Stroke risk factors, genetics, and prevention. Circ Res. 2017;120(3):472-495. doi:10.1161/CIRCRESAHA.116.308398 28154098 PMC5321635

[13] O’Donnell MJ Xavier D Liu L , et al. Risk factors for ischaemic and intracerebral haemorrhagic stroke in 22 countries (the INTERSTROKE study): a case-control study. Lancet. 2010;376(9735):112-123. doi:10.1016/S0140-6736(10)60834-3 20561675

[14] Rundek T Tolea M Ariko T Fagerli EA Camargo CJ . Vascular cognitive impairment (VCI). Neurotherapeutics. 2022;19(1):68-88. doi:10.1007/s13311-021-01170-y 34939171 PMC9130444

[15] Chang Wong E Chang CH . Vascular cognitive impairment and dementia. Contin Lifelong Learn Neurol. 2022;28(3):750-780. doi:10.1212/CON.0000000000001124 PMC9833847 35678401

[16] Pikula A Gulati M Bonnet JP , et al. Promise of lifestyle medicine for heart disease, diabetes mellitus, and cerebrovascular diseases. Mayo Clin Proc Innov Qual Outcomes. 2024;8(2):151-165. doi:10.1016/j.mayocpiqo.2023.11.005 38434935 PMC10907160

[17] Ngandu T Lehtisalo J Solomon A , et al. A 2 year multidomain intervention of diet, exercise, cognitive training, and vascular risk monitoring versus control to prevent cognitive decline in at-risk elderly people (FINGER): a randomised controlled trial. Lancet. 2015;385(9984):2255-2263. doi:10.1016/S0140-6736(15)60461-5 25771249

[18] Rees K Takeda A Martin N , et al. Mediterranean-style diet for the primary and secondary prevention of cardiovascular disease. Cochrane Database Syst Rev. 2019;385:CD009825 Heart Group Cochrane, ed., 2019. doi:10.1002/14651858.CD009825.pub3 PMC6414510 30864165

[19] Lianov L Johnson M . Physician competencies for prescribing lifestyle medicine. JAMA. 2010;304(2):202-203. doi:10.1001/jama.2010.903 20628134

[20] Rippe JM . Lifestyle medicine: the health promoting power of daily habits and practices. Am J Lifestyle Med. 2018;12(6):499-512. doi:10.1177/1559827618785554 30783405 PMC6367881

[21] Rippe JM . Lifestyle strategies for risk factor reduction, prevention, and treatment of cardiovascular disease. Am J Lifestyle Med. 2019;13(2):204-212. doi:10.1177/1559827618812395 30800027 PMC6378495

[22] Yvonne Buowari D . The role of lifestyle medicine in the management of diabetes mellitus. In: Pantea Stoian A , ed. Type 2 Diabetes - from Pathophysiology to Cyber Systems. London, UK: IntechOpen; 2021. doi:10.5772/intechopen.99555

[23] Rutten-Jacobs LC Larsson SC Malik R , et al. Genetic risk, incident stroke, and the benefits of adhering to a healthy lifestyle: cohort study of 306 473 UK Biobank participants. BMJ. 2018;24:k4168. doi:10.1136/bmj.k4168, Published online October. PMC6199557 30355576

[24] Lloyd-Jones DM Allen NB Anderson CAM , et al. Life’s essential 8: updating and enhancing the American heart association’s construct of cardiovascular health: a presidential advisory from the American heart association. Circulation. 2022;146(5):e18-e43. doi:10.1161/CIR.0000000000001078 35766027 PMC10503546

[25] Kleindorfer DO Towfighi A Chaturvedi S , et al. 2021 guideline for the prevention of stroke in patients with stroke and transient ischemic attack: a guideline from the American heart association/American stroke association. Stroke. 2021;52(7):e364-e467. doi:10.1161/STR.0000000000000375 34024117

[26] Heart and Stroke Foundation of Canada . Canadian stroke best practice recommendations: secondary prevention of stroke. 2021. https://www.strokebestpractices.ca/-/media/1-stroke-best-practices/secondary-prevention-of-stroke/csbpr7secondarypreventionevidencetable2alifestyleandriskfactormanagementdietfinal10january2021.pdf?rev=7e9c241bf1de4b3a971464052176c554. Accessed 11 July 2024.

[27] Kulik NL Moore EW Centeio EE , et al. Knowledge, attitudes, self-efficacy, and healthy eating behavior among children: results from the building healthy communities trial. Health Educ Behav. 2019;46(4):602-611. doi:10.1177/1090198119826298 30791715

[28] Pallangyo P Mkojera ZS Komba M , et al. Public knowledge of risk factors and warning signs of heart attack and stroke. Egypt J Neurol Psychiatry Neurosurg. 2024;60(1):12. doi:10.1186/s41983-023-00780-x

[29] Kitakata H Kohno T Kohsaka S , et al. Patient confidence regarding secondary lifestyle modification and knowledge of ‘heart attack’ symptoms following percutaneous revascularisation in Japan: a cross-sectional study. BMJ Open. 2018;8(3):e019119. doi:10.1136/bmjopen-2017-019119 PMC5857652 29549203

[30] Arlinghaus KR Johnston CA . Advocating for behavior change with education. Am J Lifestyle Med. 2018;12(2):113-116. doi:10.1177/1559827617745479 30283247 PMC6124997

[31] Schulz PJ Pessina A Hartung U Petrocchi S . Effects of objective and subjective health literacy on patients’ accurate judgment of health information and decision-making ability: survey study. J Med Internet Res. 2021;23(1):e20457. doi:10.2196/20457 33475519 PMC7861996

[32] Mather M Pettigrew LM Navaratnam S . Barriers and facilitators to clinical behaviour change by primary care practitioners: a theory-informed systematic review of reviews using the theoretical domains framework and behaviour change wheel. Syst Rev. 2022;11(1):180. doi:10.1186/s13643-022-02030-2 36042457 PMC9429279

[33] Gorelick PB Furie KL Iadecola C , et al. Defining optimal brain health in adults: a presidential advisory from the American heart association/American stroke association. Stroke. 2017;48(10):e284-e303. doi:10.1161/STR.0000000000000148 28883125 PMC5654545

[34] Von Elm E Altman DG Egger M Pocock SJ Gøtzsche PC Vandenbroucke JP . The Strengthening the Reporting of Observational Studies in Epidemiology (STROBE) statement: guidelines for reporting observational studies. J Clin Epidemiol. 2008;61(4):344-349. doi:10.1016/j.jclinepi.2007.11.008 18313558

[35] Singh S Oreskovic T Carr S , et al. The predictive validity of A brain care score for dementia and stroke: data from the UK biobank cohort. Front Neurol. 2023;14:14-2023. doi:10.3389/fneur.2023.1291020 PMC10725202 38107629

[36] Singh SD Gutierrez-Martinez L Newhouse A Sonni A Chemali Z Rosand J . Brain health begins with brain care. Lancet Neurol. 2022;21(11):961-962. doi:10.1016/S1474-4422(22)00397-0 36270304

[37] American College of Lifestyle Medicine . Validated tools. https://lifestylemedicine.org/project/lifestyle-medicine-assessment-tools/. Accessed 13 March 2022.

[38] Healthy Life Stars . Activities to steps conversion chart. https://wellness.osu.edu/sites/default/files/documents/2021/02/2021.02.-HLS-Step-Conversion-Chart.pdf. Accessed 2 June 2024.

[39] Poraj-Weder M Pasternak A Szulawski M . The development and validation of the health behavior motivation scale. Front Psychol. 2021;12:706495. doi:10.3389/fpsyg.2021.706495 34539508 PMC8446656

[40] IBM Corp . IBM SPSS Statistics for Windows, Version 29. Armonk, NY: IBM Corp; 2022.

[41] R Core Team . R: a language and environment for statistical computing. 2022. https://www.R-project.org

[42] Müller-Nordhorn J Nolte CH Rossnagel K , et al. Knowledge about risk factors for stroke. Stroke. 2006;37(4):946-950. doi:10.1161/01.STR.0000209332.96513.82 16514090

[43] Heger I Deckers K van Boxtel M , et al. Dementia awareness and risk perception in middle-aged and older individuals: baseline results of the MijnBreincoach survey on the association between lifestyle and brain health. BMC Public Health. 2019;19(1):678. doi:10.1186/s12889-019-7010-z 31159779 PMC6545627

[44] Glynn RW Shelley E Lawlor BA . Public knowledge and understanding of dementia—evidence from a national survey in Ireland. Age Ageing. 2017;46(5):865-869. doi:10.1093/ageing/afx082 28531240

[45] Budin-Ljøsne I Mowinckel AM Friedman BB , et al. Public perceptions of brain health: an international, online cross-sectional survey. BMJ Open. 2022;12(4):e057999. doi:10.1136/bmjopen-2021-057999 PMC9016409 35437254

[46] McFadden D. Quantitative methods for analyzing travel behaviour of individuals: some recent developments. Cowles Found Discuss Pap. 1977;707:1-47. https://elischolar.library.yale.edu/cowles-discussion-paper-series/707. Accessed 12 February 2025.

[47] American Heart Association . Target heart rates chart. 2024. https://www.heart.org/en/healthy-living/fitness/fitness-basics/target-heart-rates. Accessed 19 August 2024.

[48] Bodai BI Nakata TE Wong WT , et al. Lifestyle medicine: a brief review of its dramatic impact on health and survival. Perm J. 2018;22(1):17-025. doi:10.7812/TPP/17-025 PMC5638636 29035175

[49] Wong WP Yeung M Loh S , et al. Stroke-related knowledge, lifestyle behaviours and health beliefs in Singaporean Chinese: implications for health education. Health Educ J. 2013;72(4):386-397. doi:10.1177/0017896912446554

[50] Liu Y Wang H Bai B , et al. Trends in unhealthy lifestyle factors among adults with stroke in the United States between 1999 and 2018. J Clin Med. 2023;12(3):1223. doi:10.3390/jcm12031223 36769871 PMC9917618

[51] Zhou Y Hua B Shi X Du S Yuan J Wang Y . Exercise intention and its associated factors among persons post-stroke: a cross-sectional study. Patient Prefer Adherence. 2023;17:2535-2544. doi:10.2147/PPA.S424595 37849617 PMC10578170

[52] Faiz KW Labberton AS Thommessen B Rønning OM Barra M . Stroke-related knowledge and lifestyle behavior among stroke survivors. J Stroke Cerebrovasc Dis. 2019;28(11):104359. doi:10.1016/j.jstrokecerebrovasdis.2019.104359 31495671

[53] Althomali M Liberatos P Mubaraki AA Alqasim MA . Understanding risks for stroke and the importance of a healthy lifestyle among stroke patients at a tertiary hospital in Saudi Arabia. Saudi J Health Sci. 2024;13(1):56-77. doi:10.4103/sjhs.sjhs_108_23

[54] Bukhari S Yaghi S Bashir Z . Stroke in young adults. J Clin Med. 2023;12(15):4999. doi:10.3390/jcm12154999 37568401 PMC10420127

[55] American Heart Association . American Heart Association. 100 Years Bold Heart. Healthy Living. https://www.heart.org/en/healthy-living. Accessed 6 July 2024.

[56] World Stroke Organization . The world stroke campaign. 2024. https://www.world-stroke.org/world-stroke-day-campaign. Accessed 6 July 2024.

[57] Hsu MSH Rouf A Allman-Farinelli M . Effectiveness and behavioral mechanisms of social media interventions for positive nutrition behaviors in adolescents: a systematic review. J Adolesc Health. 2018;63(5):531-545. doi:10.1016/j.jadohealth.2018.06.009 30197198

[58] Chau MM Burgermaster M Mamykina L . The use of social media in nutrition interventions for adolescents and young adults—a systematic review. Int J Med Inf. 2018;120:77-91. doi:10.1016/j.ijmedinf.2018.10.001 PMC6983924 30409348

[59] Lecouturier J Rodgers H Murtagh MJ White M Ford GA Thomson RG . Systematic review of mass media interventions designed to improve public recognition of stroke symptoms, emergency response and early treatment. BMC Public Health. 2010;10(1):784. doi:10.1186/1471-2458-10-784 21182777 PMC3022856

[60] Goodyear V Armour K . Young People, Social Media and Health London. Oxfordshire, UK: Routledge; 2019.

[61] World Health Organization . Global Action Plan on Physical Activity 2018–2030: More Active People for a Healthier World. Geneva, Switzerland: World Health Organization; 2018. https://iris.who.int/handle/10665/272722. Accessed 6 July 2024.

[62] Cockerham WC . Healthy Lifestyles: Bringing Structure Back. Hoboken, NJ: The Wiley Blackwell Companion to Medical Sociology; 2021.

[63] Nedjar-Guerre A Wattelez G Serra-Mallol C Frayon S Galy O . Adolescent screen time and unhealthy food consumption in the context of the digital development in New Caledonia. PLoS One. 2023;18:1-15. doi:10.1371/journal.pone.0285374 PMC10166542 37155631

[64] Zink J Booker R Wolff-Hughes DL , et al. Longitudinal associations of screen time, physical activity, and sleep duration with body mass index in U.S. youth. Int J Behav Nutr Phys Activ. 2024;21(1):35. doi:10.1186/s12966-024-01587-6 PMC10988901 38566134

[65] Ørtenblad L Høtoft D Krogh RH , et al. Women’s perspectives on motivational factors for lifestyle changes after gestational diabetes and implications for diabetes prevention interventions. Endocrinol Diabetes Metab. 2021;4(3):e00248. doi:10.1002/edm2.248 34277972 PMC8279634

[66] Enjezab B Farajzadegan Z Taleghani F Aflatoonian A . Internal motivations and barriers effective on the healthy lifestyle of middle-aged women: a qualitative approach. Iran J Nurs Midwifery Res. 2023;17(5):390-398. PMC3703082 23853654

[67] Deijle IA Van Schaik SM Van Wegen EEH Weinstein HC Kwakkel G Van Den Berg-Vos RM . Lifestyle interventions to prevent cardiovascular events after stroke and transient ischemic attack: systematic review and meta-analysis. Stroke. 2017;48(1):174-179. doi:10.1161/STROKEAHA.116.013794 27924055

[68] Hall P Lawrence M Blake C Lennon O . Interventions for behaviour change and self-management of risk in stroke secondary prevention: an overview of reviews. Cerebrovasc Dis. 2024;53(1):1-13. doi:10.1159/000531138 37231867

[69] Fallows ES . Lifestyle medicine: a cultural shift in medicine that can drive integration of care. Future Healthc J. 2023;10(3):226-231. doi:10.7861/fhj.2023-0094 38162213 PMC10753218

[70] Agarwal S Wade AN Mbanya JC , et al. The role of structural racism and geographical inequity in diabetes outcomes. Lancet. 2023;402(10397):235-249. doi:10.1016/S0140-6736(23)00909-1 37356447 PMC11329296

[71] Towfighi A Boden-Albala B Cruz-Flores S , et al. Strategies to reduce racial and ethnic inequities in stroke preparedness, care, recovery, and risk factor control: a scientific statement from the American heart association. Stroke. 2023;54(7):e371-e388. doi:10.1161/STR.0000000000000437 37183687

[72] Mensah GA Cooper RS Siega-Riz AM , et al. Reducing cardiovascular disparities through community-engaged implementation research: a national heart, lung, and blood institute workshop report. Circ Res. 2018;122(2):213-230. doi:10.1161/CIRCRESAHA.117.312243 29348251 PMC5777283

[73] Sarfo FS Ovbiagele B . Utilizing implementation science to bridge cerebrovascular health disparities: a local to global perspective. Curr Neurol Neurosci Rep. 2022;22(5):293-303. doi:10.1007/s11910-022-01193-8 35381952 PMC9081275

[74] Yoshikawa A Smith ML Lee S Towne SD Ory MG . The role of improved social support for healthy eating in a lifestyle intervention: *texercise Select*. Public Health Nutr. 2021;24(1):146-156. doi:10.1017/S1368980020002700 32830625 PMC10195600

[75] Dysinger WS . Lifestyle medicine prescriptions. Am J Lifestyle Med. 2021;15(5):555-556. doi:10.1177/15598276211006627 34646106 PMC8504327

[76] Dysinger WS . Lifestyle medicine competencies for primary care physicians. AMA J Ethics. 2013;15(4):306-310. doi:10.1001/virtualmentor.2013.15.4.medu1-1304 23566779

[77] Tack RWP Senff JR Kimball TN , et al. Reliability and validity of self‐reported risk factors for stroke and dementia. J Am Heart Assoc. 2025;14:e038730. doi:10.1161/JAHA.124.038730 40118792 PMC12132855

