# Supplementary material for: Lifestyle Knowledge and Behavior Among Stroke and High-Risk Younger Adult Patients Through Sex, Age and Stroke Status Differences: A Cross-Sectional Study
Source: Am J Lifestyle Med. 2025 May 23:15598276251343016. Online ahead of print. doi: 10.1177/15598276251343016 (PMC12102088; doi:10.1177/15598276251343016)
Supplement: Supplemental Material - Lifestyle Knowledge and Behavior Among Stroke and High-Risk Younger Adult Patients Through Sex, Age and Stroke Status Differences: A Cross-Sectional Study [file sj-pdf-1-ajl-10.1177_15598276251343016.pdf]

Supplemental Table 1. Sociodemographic Characteristics

	**Below 45 years** (n=43)	**Above 45 years** (n=61)	**Total** (N=104)	**p-value**
**Sex (n,%)**				0.233(1)
Female	26(60.5%)	29(47.5%)	55(52.9%)	
Male	17(39.5%)	32(52.5%)	49(47.1%)	
**Gender (n,%)**				0.194(1)
Men	17(39.5%)	32(52.5%)	49(47.1%)	
Women	25(58.1%)	29(47.5%)	54(51.9%)	
Non-binary	1(2.3%)	0(0.0%)	1(1.0%)	
**Ethnicity (n,%)**				0.213(1)
Missing	6	7	13	
White	17(45.9%)	37(68.5%)	54(59.3%)	
European	2(5.4%)	1(1.9%)	3(3.3%)	
Black	3(8.1%)	1(1.9%)	4(4.4%)	
South Asian	2(5.4%)	2(3.7%)	4 (4.4%)	
East Asian	5(13.5%)	7(13.0%)	12(13.2 %)	
Mainland Southeast Asian	0(0.0%)	1(1.9%)	1(1.1%)	
West Asian	0(0.0%)	1(1.9%)	1(1.1%)	
Latin American	5(13.5%)	2(3.7%)	7(7.7%)	
Arab	1(2.7%)	0(0.0%)	1(1.1%)	
West Indian	1(2.7%)	1(1.9%)	2(2.2%)	
African	0(0.0%)	1(1.9%)	1(1.1%)	
Prefer Not to Answer	1(2.7%)	0(0.0%)	1(1.1%)	
**Canadian or Foreign Born** (n,%)				0.559(1)
Missing	6	7	13	
Canadian-Born	24(64.9%)	34(63.0%)	58(63.7%)	
Foreign-Born	12(32.4%)	20(37.0%)	32(35.2%)	
Do not wish to answer	1(2.7%)	0(0.0%)	1(1.1%)	
**Language** (n,%)				0.488(1)
Missing	6	7	13	
English first language	24(64.9%)	41(75.9%)	65(71.4%)	
English second language	12(32.4%)	12(22.2%)	24(26.4%)	
Do not wish to answer	1(2.7%)	1(1.9%)	2(2.2%)	
**Marital Status** (n,%)				**0.003(1)**
Missing	6	7	13	
Single	19(51.4%)	10(18.5%)	29 (31.9%)	
Married/Cohabitating	17(45.9%)	41(75.9%)	58(63.7%)	
Other	1(2.7%)	3(5.6%)	4(4.4%)	
**Level of Education** (n,%)				0.605(1)
Missing	7	7	14	
No degree, certificate, or diploma	1(2.8%)	2(3.7%)	3 (3.3%)	
High school	4(11.1%)	8(14.8%)	12 (13.3%)	

	**Below 45 years** (n=43)	**Above 45 years** (n=61)	**Total** (N=104)	**p-value**
Some college/university	2(5.6%)	5(9.3%)	7 (7.8%)	
College/university	19(52.8%)	24(44.4%)	43 (47.8%)	
Registered apprenticeship/ trade certificate	2(5.6%)	0(0.0%)	2(2.2%)	
Post graduate degree	8(22.2%)	15(27.8%)	23 (25.6%)	
**Employment Type** (n,%)				0.121(1)
Missing	7	7	14	
Manual paid work	6(16.7%)	15(27.8%)	21 (23.3%)	
Non-manual paid work	11(30.6%)	14(25.9%)	25 (27.8%)	
Student	6(16.7%)	1(1.9%)	7 (7.8%)	
Homemaker	0 (0.0%)	2(3.7%)	2 (2.2%)	
Unpaid Volunteer	0(0.0%)	1(1.9%)	1 (1.1%)	
Unemployed, looking for work	5(13.9%)	3(5.6%)	8 (8.9%)	
On leave of absence	6(16.7%)	8(14.8%)	14 (15.6%)	
Retired	1(2.8%)	5(9.3%)	6 (6.7%)	
Disability	1(2.8%)	3(5.6%)	4 (4.4%)	
Not working due to illness/injury	0(0.0%)	2(3.7%)	2(2.2%)	
**Average Income** (n,%)				**0.001(1)**
Missing	6	7	13	
Less than $15,000	0(0.0%)	5(9.3%)	5(5.5%)	
$15,000-$49,999	7(18.9%)	3(5.6%)	10(11.0%)	
$50,000-$99,999	8(21.6%)	18(33.3%)	26(28.6%)	
$100,000-$200,000	7(18.9%)	21(38.9%)	28(30.8%)	
More than $200,000	10(27.0%)	3(5.6%)	13(14.3%)	
Do not know	2(5.4%)	0(0.0%)	2(2.2%)	
Do not wish to answer	3(8.1%)	4(7.4%)	7(7.7%)	

1. Pearson's Chi-squared test

Supplemental Table 2. Lifestyle adoption around Brain Health

	**Female (n=55)**	**Male (n=49)**	**Total (N=104)**	**p-value**
**Willingness to change lifestyle in next 6 months to improve health** (n,%)				0.677 (1)
Missing	0	1	1	
Extremely willing	27 (49.1%)	21 (43.8%)	48 (46.6%)	
Likely willing	17 (30.9%)	20 (41.7%)	37 (35.9%)	
Somewhat willing	10 (18.2%)	6 (12.5%)	16 (15.5%)	
Not at all willing	1 (1.8%)	1 (2.1%)	2 (1.9%)	

	Female (n=55)	Male (n=49)	Total (N=104)	p-value
**Primary priority to improve health in next 6 months** (n,%)				0.530 (1)
Missing	23	18	41	
Exercise	9 (28.1%)	8 (25.8%)	17 (27.0%)	
Substance Use	1 (3.1%)	3 (9.7%)	4 (6.3%)	
Purpose & Connection	4 (12.5%)	1 (3.2%)	5 (7.9%)	
Nutrition	8 (25.0%)	10 (32.3%)	18 (28.6%)	
Sleep	7 (21.9%)	8 (25.8%)	15 (23.8%)	
Mental Health	3 (9.4%)	1 (3.2%)	4 (6.3%)	
**Secondary priority to improve health in next 6 months** (n,%)				0.676 (1)
Missing	23	18	41	
Exercise	9 (28.1%)	10 (32.3%)	19 (30.2%)	
Substance Use	0 (0.0%)	1 (3.2%)	1 (1.6%)	
Purpose & Connection	3 (9.4%)	5 (16.1%)	8 (12.7%)	
Nutrition	8 (25.0%)	8 (25.8%)	16 (25.4%)	
Sleep	7 (21.9%)	5 (16.1%)	12 (19.0%)	
Mental Health	5 (15.6%)	2 (6.5%)	7 (11.1%)	
**Tertiary priority to improve health in next 6 months** (n,%)				0.275 (1)
Missing	23	18	41	
Exercise	5 (15.6%)	6 (19.4%)	11 (17.5%)	
Substance Use	0 (0.0%)	1 (3.2%)	1 (1.6%)	
Purpose & Connection	4 (12.5%)	5 (16.1%)	9 (14.3%)	
Nutrition	11 (34.4%)	3 (9.7%)	14 (22.2%)	
Sleep	6 (18.8%)	8 (25.8%)	14 (22.2%)	
Mental Health	6 (18.8%)	8 (25.8%)	14 22.2 %)	

1. Pearson's chi-squared test

Supplemental Table 3. Health Behaviour Motivation Scale (HBMS)

	Female (n=55)	Male (n=49)	Total (N=104)	p-value
**Engage in healthy lifestyle because makes one happy** (n,%)				0.104 (1)
Missing	1	2	3	
Does not suit me at all	7 (13.0%)	1 (2.1%)	8 (7.9%)	

	Female (n=55)	Male (n=49)	Total (N=104)	p-value
Suits me very poorly	6 (11.1%)	3 (6.4%)	9 (8.9%)	
Suits me on average	12 (22.2%)	14 (29.8%)	26 (25.7%)	
Suits me well	12 (22.2%)	18 (38.3%)	30 (29.7%)	
Suits me very well	17 (31.5%)	11 (23.4%)	28 (27.7%)	
**Engage in healthy lifestyle because is an important & ongoing task** (n,%)				0.189 (1)
Missing	1	2	3	
Does not suit me at all	2 (3.7%)	2 (4.3%)	4 (4.0%)	
Suits me very poorly	4 (7.4%)	0 (0.0%)	4 (4.0%)	
Suits me on average	6 (11.1%)	7 (14.9%)	13 (12.9%)	
Suits me well	15 (27.8%)	20 (42.6%)	35 (34.7%)	
Suits me very well	27 (50.0%)	18 (38.3%)	45 (44.6%)	
**Engage in healthy lifestyle because of guilt or remorse if health is neglected** (n,%)				0.063 (1)
Missing	1	2	3	
Does not suit me at all	10 (18.5%)	10 (21.3%)	20 (19.8%)	
Suits me very poorly	6 (11.1%)	11 (23.4%)	17 (16.8%)	
Suits me on average	10 (18.5%)	13 (27.7%)	23 (22.8%)	
Suits me well	17 (31.5%)	11 (23.4%)	28 (27.7%)	
Suits me very well	11 (20.4%)	2 (4.3%)	13 (12.9%)	
**Engage in healthy lifestyle to make others happy** (n,%)				0.759 (1)
Missing	2	2	4	
Does not suit me at all	15 (28.3%)	12 (25.5%)	27 (27.0%)	
Suits me very poorly	13 (24.5%)	8 (17.0%)	21 (21.0%)	
Suits me on average	10 (18.9%)	13 (27.7%)	23 (23.0%)	
Suits me well	10 (18.9%)	8 (17.0%)	18 (18.0%)	
Suits me very well	5 (9.4%)	6 (12.8%)	11 (11.0%)	

	Female (n=55)	Male (n=49)	Total (N=104)	p-value
**Engage in healthy lifestyle because others expect me to take care of my health** (n,%)				0.903 (1)
Missing	2	2	4	
Does not suit me at all	11 (20.8%)	11 (23.4%)	22 (22.0%)	
Suits me very poorly	6 (11.3%)	4 (8.5%)	10 (10.0%)	
Suits me on average	15 (28.3%)	15 (31.9%)	30 (30.0%)	
Suits me well	11 (20.8%)	11 (23.4%)	22 (22.0%)	
Suits me very well	10 (18.9%)	6 (12.8%)	16 (16.0%)	
**Don't engage in a healthy lifestyle because a feeling of helplessness arises in me** (n,%)				0.891 (1)
Missing	1	2	3	
Statement does not suit me at all	28 (51.9%)	27 (57.4%)	55 (54.5%)	
Statement suits me very poorly	11 (20.4%)	6 (12.8%)	17 (16.8%)	
Statement suits me on average	7 (13.0%)	7 (14.9%)	14 (13.9%)	
Statement suits me well	5 (9.3%)	4 (8.5%)	9 (8.9%)	
Statement suits me very well	3 (5.6%)	3 (6.4%)	6 (5.9%)	

1. Pearson's chi-squared test
